# Synthesis and biological evaluation of novel benzyl piperazine derivatives of 5-(5-nitroaryl)-1,3,4-thiadiazoles as Anti-*Helicobacter pylo*ri agents

**DOI:** 10.1186/2008-2231-21-66

**Published:** 2013-08-08

**Authors:** Negar Mohammadhosseini, Parastoo Saniee, Ameneh Ghamaripour, Hassan Aryapour, Farzaneh Afshar, Najmeh Edraki, Farideh Siavoshi, Alireza Foroumadi, Abbas Shafiee

**Affiliations:** 1 Department of Medicinal Chemistry, Faculty of Pharmacy and Pharmaceutical Sciences Research Center, Tehran University of Medical Sciences, Tehran 14176, Iran; 2 Department of Microbiology, Faculty of Sciences, University of Tehran, P.O. Box: 14155–6455, Tehran, Iran; 3 Department of Biology, Faculty of Science, Golestan University, Gorgan, Iran; 4 Department of Chemistry, Science and Research Branch, Islamic Azad University, Arak, Iran

**Keywords:** Anti-*Helicobacter pylori* activity, 1,3,4-Thiadiazole, Nitrofuran, Nitrothiophen

## Abstract

**Background and the purpose of the study:**

*Helicobacter pylori* is recognized as the main cause of gastritis and gastroduodenal ulcers and classified as class 1 carcinogen pathogen. Different 1,3,4-thiadiazole derivatives bearing 5-nitroaryl moiety have been shown considerable anti- *H. pylori* activity. In attempt to find new and potent derivatives of described scaffold, a new series of 1-(substituted benzyl)-4-(5-(5-nitroaryl-2-yl)-1,3,4-thiadiazol-2-yl)piperazine derivatives were synthesized and evaluated against three metronidazole-resistant isolates of *H. pylori* using paper disk diffusion bioassay test.

**Methods:**

The title compounds were prepared through the reaction of 1-(5-(5-nitroaryl-2-yl)-1,3,4-thiadiazol-2-yl) piperazine **5a-b** and substituted benzyl chloride in DMF. The inhibitory activity of the new derivatives **6a-q** against three metronidazole-resistant isolates of *H. pylori* was evaluated by the disc diffusion method and compared with the commercially available standard drug metronidazole.

**Results and discussion:**

The results of SAR study indicated that the potency and anti-*H. pylori* activity profile of synthesized derivatives is mainly attributed to the substituted nitroaryl moiety at the C-5 position of 1,3,4-thiadiazole ring. Most of 1,3,4-thiadiazole derivatives bearing 5-nitrofuran moiety at C-5 position of central thiadiazole ring, demonstrated more promising anti-*H. pylori* than the 5-nitrothiophen counterpart.

**Conclusion:**

The most potent nitrofuran derivative containing 3-methoxybenzyl piperazine pendant at the C-2 position of 1,3,4-thiadiazole ring (compound **6i)**, demonstrated strong anti-*H. pylori* potential at studied concentrations 100-25 μg/disk (IZD > 20 mm) against all studied metronidazole- resistant isolates of *H. pylori*.

## Introduction

*Helicobacter pylori,* an spiral-shaped Gram-negative bacterium, has been considered as the leading cause of gastritis and gastroduodenal ulcer in developing countries. *H. pylori* is also classified as the class 1 carcinogen pathogen because of its epidemiological relationship to gastric adenocarcinoma and gastric mucosa-associated lymphoid tissue lymphoma [1-3]. Therefore; treatment of *Helicobacter pylori* requires targeted therapeutic strategy.

Different studies show that eradication of *H. pylori* infection resulted to ulcer healing and reduced prevalence of gastric cancer [4]. However, treatment of this infection is complicated and successful eradication of this organism is continuously requiring a combination regime using a minimum of two different antibiotics plus proton pump inhibitor (PPI) agent [3,5,6].

Although several combination therapy regimes using various anti-bacterial agents through different duration of therapy are proposed for eradication of *H. pylori* infection, emergence of resistance strains is a growing global concern. Clinical evaluation of current therapeutic agents indicated the incidence of drug-drug interaction, infection relapses and side effects of common drugs [7,8]. These factors have been the rationale for the development of new anti- *Helicobacter pylori* drugs and search for novel therapeutic molecules that offer better protection and decreased relapse towards resistant strains.

**Figure 1:**
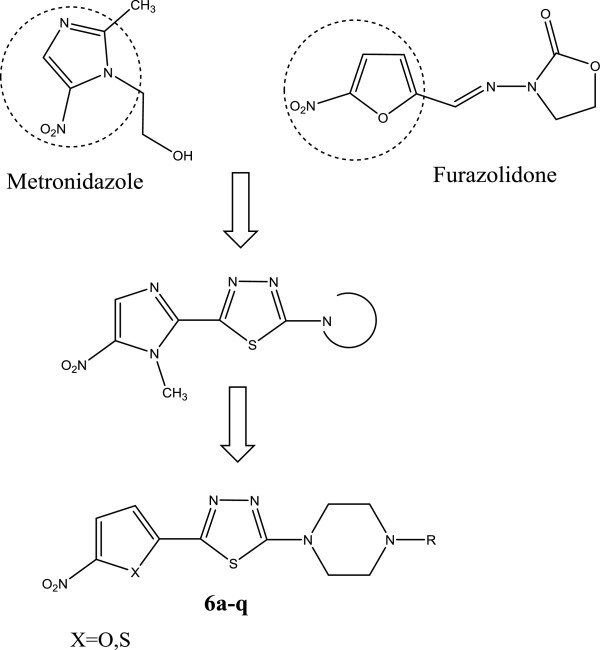
**Chemical structure of current nitroheterocyclic drugs (Metronidazole and Furazolidone) used in the treatment of *H.pylori *infection and designed 5-(nitroaryl)-1,3,4-thiadiazoles bearing piperazine derivatives 6a-q.**

Nitrofuran and nitrothiophene heterocyclic derivatives have been extensively studied in therapy against different microbial infections [9-11]. Moreover, the antimicrobial and anti-Helicobacter property of 1,3,4-thiadiazole moiety is well established and attachment of this antimicrobial scaffold with nitro-hetrocyclic moieties would accommodate the bioresponses and antimicrobial activity depending on the type of substituted group and position of attachment [12-14]. In our previous works, we have investigated the anti-Helicobacter potential of different 5-(5-nitroaryl)-1,3,4-thiadiazole scaffold bearing different C-2 attached pendants. Among different nitroheterocycles, 5-nitrofuran, 5-nitrothiophen and 5-nitroimidazole moieties are preferable for substitution at C-5 position of 1,3,4-thiadiazole ring. These nitroheteroaromatic moieties mimic the nitroaromatic part of nitroheterocyclic drugs such as metronidazole and furazolidon (Figure 1) [11,14-17]. In continuation of our research program to find a novel antibacterial agent [18], we have previously demonstrated the considerable antibacterial activity of 5-nitroimidazole-based 1,3,4-thiadiazoles bearing cyclic amine functionality such as pyrrolidine and piperazine derivatives at the C-2 position of thiadiazole ring against resistant strains of *Helicobacter pylori*[15]. In order to find the structural requirement of cyclic amine derivatives of 5-(nitroaryl)-1,3,4-thiadiazole as anti*-H. pylori* agents, herein, we describe the synthesis and anti-*Helicobacter* evaluation of a new series of 5-(nitrothienyl) and 5-(nitrofuryl)-1,3,4-thiadiazoles containing different piperazine side chain at 2-position of 1,3,4-thiadiazole ring system (Figure 1).

## Material and methods

### Chemistry

A Kofler hot stage apparatus was used for the measurement of reported melting. The IR spectra were recorded on a Nicollet FT-IR Magna 550 spectrometer. The ^1^H NMR spectra were recorded on a Varian FT-400 MHz or Bruker FT-500 MHz spectrometer and chemical shifts (δ) are reported in ppm relative to internal tetramethylsilane. The mass spectra were run on a Agilent 1100/Bruker Daltonic (Ion trap) VL instrument. at 70 eV. Fross- Heraeus CHN-O rapid analyzer was used for elemental analysis of synthesized compounds and the results are within ± 0.4% of the theoretical values. Analytical thin-layar chromatography (TLC) on Merck silicagel 60 F254 plates using various mobile phases of different polarities was performed in order to confirm the purity of final products.

#### General method for the synthesis of 1-substitutedbenzyl-4-(5-(5-nitroaryl-2-yl)-1,3,4-thiadiazol-2-yl)piperazine 6a-q

To a mixture of 1-(5-(5-nitroaryl-2-yl)-1,3,4-thiadiazol-2-yl)piperazine **5a-b** (1.0 mmol) and different benzyl chloride derivatives (1.0 mmol) in DMF (15 mL), NaHCO_3_ (0.21 mmol) was added and the resulted mixture was stirred at room temperature overnight. After completion of the reaction, water was added, and the resulted precipitate was filtered off, washed with water, and crystallized from ethanol.

#### 1-(2-nitrobenzyl)-4-(5-(5-nitrothiophen-2-yl)-1,3,4-thiadiazol-2-yl)piperazine (6a)

Yield 40%; m.p. 219-221°C; IR(KBr): 1343, 1527 cm^-1^ (NO_2_); ^1^H-NMR(CDCl_3_) δ: 2.48-2.64 (m, 4H, piperazine), 3.48-3.63 (m, 2H, CH_2_), 3.48-3.63 (m, 4H, piperazine), 7.16 (bs, 1H, thiophene), 7.45 (bs, 1H, phenyl), 7.52-7.60 (m, 2H, phenyl), 7.80-7.92 (m, 2H, phenyl-thiophene); MS: m/z (%) 432 (M^+^, 1), 415 (21), 241 (15), 191 (81), 172 (15), 136 (100), 120 (33), 91 (34), 69 (93), 42 (25). Anal. Calcd. For C_17_H_16_N_6_O_4_S_2_: C, 47.21; H, 3.73; N, 19.43; Found: C, 47.03; H, 3.51; N, 19.67.

#### 1-(3-nitrobenzyl)-4-(5-(5-nitrothiophen-2-yl)-1,3,4-thiadiazol-2-yl)piperazine (6b)

Yield 33%; m.p. 205-206°C; IR(KBr): 1344, 1528 cm^-1^ (NO_2_); ^1^H-NMR(CDCl_3_) δ: 2.26-2.69 (m, 4H, piperazine), 3.60-3.72 (m, 4H, piperazine and 2H, CH_2_), 7.16 (bs, 1H, thiophene), 7.58 (t, 1H, phenyl, *J* = 7.5Hz), 7.68 (bs, 1H, phenyl), 7.86 (bs, 1H, thiophene), 8.15 (bs, 1H, phenyl), 8.24 (s, 1H, phenyl); MS: m/z (%) 432 (M^+^, 2), 241 (14), 191 (88), 172 (14), 155 (16), 136 (100), 111 (16), 90 (60), 73 (18), 56 (47). Anal. Calcd. For C_17_H_16_N_6_O_4_S_2_: C, 47.21; H, 3.73; N, 19.43; Found: C, 47.54; H, 3.61; N, 19.83.

#### 1-(4-nitrobenzyl)-4-(5-(5-nitrothiophen-2-yl)-1,3,4-thiadiazol-2-yl)piperazine (6c)

Yield 60%; m.p. 232-234°C; IR(KBr): 1340, 1509 cm^-1^ (NO_2_); ^1^H-NMR(CDCl_3_) δ: 2.35-2.50 (m, 4H, piperazine), 3.40-3.60 (m, 4H, piperazine and 2H, CH_2_), 7.02 (s, 1H, thiophene), 7.37 (d, 2H, phenyl, *J* = 7.6Hz), 7.70 (s, 1H, thiophene), 8.02 (d, 2H, phenyl, *J* = 7.6 Hz); MS: m/z (%)432 (M^+^, 16), 415 (16), 395 (46), 368 (11), 313 (11), 296 (14), 241 (32), 191 (100), 172 (33), 155 (12), 136 (77), 106 (31), 78 (37), 56 (30). Anal. Calcd. For C_17_H_16_N_6_O_4_S_2_ C, 47.21; H, 3.73; N, 19.43; Found: C, 47.56; H, 3.99; N, 19.07.

#### 1-(2,6-difluorobenzyl)-4-(5-(5-nitrothiophen-2-yl)-1,3,4-thiadiazol-2-yl)piperazine (6d)

Yield 31%; m.p. 198-199°C; IR(KBr): 1343, 1503 cm^-1^ (NO_2_); ^1^H-NMR(CDCl_3_) δ: 2.64-2.76 (m, 4H, piperazine), 3.60-3.71 (m, 4H, piperazine), 3.79 (s, 2H, CH_2_), 6.92 (t, 3H, phenyl, *J* = 7.6Hz), 7.14 (d, 1H, thiophene, *J* = 3.6Hz ), 7.85 (d, 1H, thiophene, *J* = 3.6Hz); MS: m/z (%) 423 (M^+^, 2), 182 (100), 166 (30), 127 (100), 111 (15), 83 (21), 57 (31), 41 (23). Anal. Calcd. For C_17_H_15_F_2_N_5_O_2_S_2_: C, 48.22; H, 3.57; N, 16.54; Found: C, 48.49; H, 3.34; N, 16.16.

#### 1-(2,4,5-trifluorobenzyl)-4-(5-(5-nitrothiophen-2-yl)-1,3,4-thiadiazol-2-yl)piperazine (6e)

Yield 33%; m.p. 213-215°C; IR(KBr): 1342, 1513 cm^-1^ (NO_2_); ^1^H-NMR(CDCl_3_) δ: 2.64 (bs, 4H, piperazine), 3.59 (s, 2H, CH_2_), 3.65 (bs, 4H, piperazine), 6.90-7.00 (m, 1H, phenyl), 7.12 (bs, 1H, thiophene), 7.20-7.38 (m, 1H, phenyl), 7.87 (bs, 1H, thiophene); MS: m/z (%) 441 (M^+^, 5), 241 (13), 200 (86), 182 (19), 145 (100), 128 (11), 69 (11), 42 (14). Anal. Calcd. For C_17_H_14_F_3_N_5_O_2_S_2_: C, 46.25; H, 3.20; N, 15.86; Found: C, 45.88; H, 3.44; N, 16.03.

#### 1-(2,5-diChlorobenzyl)-4-(5-(5-nitrothiophen-2-yl)-1,3,4-thiadiazol-2-yl)piperazine (6f)

Yield 93%; m.p. 210-211°C; IR(KBr): 1344, 1504 cm^-1^ (NO_2_); ^1^H-NMR(CDCl_3_) δ: 2.65-2.74 (m, 4H, piperazine), 3.60-3.74 (m, 2H, CH_2_ and 4H, piperazine), 7.13-7.32 (m, 3H, phenyl-thiophene), 7.50 (s, 1H, phenyl), 7.86 (d, 1H, thiophene, *J* = 3.6 Hz); MS: m/z (%) 459 (M^+^+4, 0.4), 457 (M^+^+2, 3), 455 (M^+^, 4), 214 (93), 192 (18), 159 (100), 123 (18). Anal. Calcd. For C_17_H_15_Cl_2_N_5_O_2_S_2_: C, 44.74; H, 3.31; N, 15.35; Found: C, 44.51; H, 3.70; N, 15.67.

#### 1-(3,4-dichlorobenzyl)-4-(5-(5-nitrothiophen-2-yl)-1,3,4-thiadiazol-2-yl)piperazine (6g)

Yield 36%; m.p. 183-185°C; IR(KBr): 1343, 1508 cm^-1^ (NO_2_); ^1^H-NMR(CDCl_3_) δ: 2.51-2.63 (m, 4H, piperazine), 3.52 (s, 2H, CH_2_), 3.60-3.68 (m, 4H, piperazine), 7.13-7.24 (m, 2H, phenyl-thiophene), 7.40 (dd, 2H, phenyl), 7.85 (s, 1H, thiophene); MS: m/z (%) 459 (M^+^+4, 0.5), 457 (M^+^+2, 3), 455 (M^+^, 5), 241 (18), 214 (81), 159 (100), 124 (16), 89 (12), 56 (19). Anal. Calcd. For C_17_H_15_Cl_2_N_5_O_2_S_2_: C, 44.74; H, 3.31; N, 15.35; Found: C, 44.46; H, 3.62; N, 15.14.

#### 1-(4-bromobenzyl)-4-(5-(5-nitrothiophen-2-yl)-1,3,4-thiadiazol-2-yl)piperazine (6h)

Yield 50%; m.p. 207-208°C; IR(KBr): 1343, 1509 cm^-1^ (NO_2_); ^1^H-NMR(CDCl_3_) δ: 2.53-2.68 (m, 4H, piperazine), 3.52 (s, 2H, CH_2_), 3.60-3.69 (m, 4H, piperazine), 7.14-7.20 (m, 3H, phenyl-thiophene), 7.44-7.50 (m, 2H, phenyl), 7.85 (s, 1H, thiophene); MS: m/z (%) 467 (M^+^+2, 13), 465 (M^+^, 14), 296 (16), 280 (14), 265 (10), 254 (41), 239 (100). Anal. Calcd. For C_17_H_16_BrN_5_O_2_S_2_: C, 43.78; H, 3.46; N, 15.02; Found: C, 43.94; H, 3.74; N, 14.78.

#### 1-(3-methoxybenzyl)-4-(5-(5-nitrofuran-2-yl)-1,3,4-thiadiazol-2-yl)piperazine (6i)

Yield 54%; m.p. 126-127°C; IR(KBr): 1355, 1536 cm^-1^ (NO_2_); ^1^H-NMR(CDCl_3_) δ: 2.63 (t, 4H, piperazine, *J* = 5.1 Hz), 3.57 (s, 2H, CH_2_), 3.67 (t, 4H, piperazine, *J* = 5.1Hz), 3.83 (s, 3H, OCH_3_), 6.84-6.87 (m, 1H, phenyl), 6.92-6.95 (m, 2H, phenyl), 7.17 (d, 1H, furan, *J* = 3.7 Hz), 7.21-7.23 (m, 1H, phenyl), 7.44 (d, 1H, furan, *J* = 3.7 Hz); MS: m/z (%) 401 (M^+^, 7), 176 (86), 147 (13), 121 (100), 91 (24), 57 (18), 40 (14). Anal. Calcd. For C_18_H_19_N_5_O_4_S: C, 53.85; H, 4.77; N, 17.45; Found: C, 53.97; H, 4.54; N, 17.79.

#### 1-(2-nitrobenzyl)-4-(5-(5-nitrofuran-2-yl)-1,3,4-thiadiazol-2-yl)piperazine (6j)

Yield 43%; m.p. 197-199°C; IR(KBr): 1353,1529 cm^-1^ (NO_2_); ^1^H-NMR(CDCl_3_) δ: 2.62 (t, 4H, piperazine, *J* = 5.1 Hz), 3.62 (t, 4H, piperazine, *J* = 5.1 Hz), 3.89 (s, 2H, CH_2_), 7.17 (d, 1H, furan, *J* = 3.8 Hz), 7.44 (d, 1H, furan, *J* = 3.8 Hz),7.45-7.48 (m, 1H, phenyl), 7.56-7.59 (m, 2H, phenyl), 7.85 (d, 1H, phenyl, *J* = 7.8 Hz); MS: m/z (%) 416 (M^+^, 2), 399 (46), 381 (13), 225 (18), 191 (86), 166 (14), 136 (100), 105 (12), 78 (44), 56 (19). Anal. Calcd. For C_17_H_16_N_6_O_5_S:C, 49.03; H, 3.87; N, 20.18; Found: C, 49.28; H, 3.64; N, 20.42.

#### 1-(3-nitrobenzyl)-4-(5-(5-nitrofuran-2-yl)-1,3,4-thiadiazol-2-yl)piperazine (6k)

Yield 50%; m.p. 144-146°C; IR(KBr): 1353, 1529 cm^-1^ (NO_2_); ^1^H-NMR(CDCl_3_) δ: 2.66 (t, 4H, piperazine, *J* = 5.0 Hz), 3.69 (t, 4H, piperazine, *J* = 5.0 Hz), 3.71 (s, 2H, CH_2_), 7.18 (d, 1H, furan, *J* = 3.9 Hz), 7.44 (d, 1H, furan, *J* = 3.9 Hz ), 7.54 (t, 1H, phenyl, *J* = 7.9 Hz), 7.71 (d, 1H, phenyl, *J* = 7.9 Hz), 8.17 (d, 1H, phenyl, *J* = 7.9 Hz), 8.26 (bs, 1H, phenyl); MS: m/z (%) 416 (M^+^, 7), 399 (12), 225 (19), 191 (100), 166 (20), 136 (86), 90 (37), 57 (21), 40 (18). Anal. Calcd. For C_17_H_16_N_6_O_5_S: C, 49.03; H, 3.87; N, 20.18; Found: C, 48.88; H, 3.56; N, 20.44.

#### 1-(4-nitrobenzyl)-4-(5-(5-nitrofuran-2-yl)-1,3,4-thiadiazol-2-yl)piperazine (6l)

Yield 50%; m.p. 212-213°C; IR(KBr): 1348, 1505 cm^-1^ (NO_2_); ^1^H-NMR(CDCl_3_) δ: 2.65 (t, 4H, piperazine, *J* = 5.1 Hz), 3.69(s, 2H, CH_2_), 3.70 (t, 4H, piperazine, *J* = 5.1 Hz), 7.18 (d, 1H, furan, *J* = 3.9 Hz), 7.44 (d, 1H, furan, *J* = 3.9 Hz), 7.56 (d, 2H, phenyl, *J* = 8.6 Hz), 8.23 (d, 2H, phenyl, *J* = 8.6 Hz); MS: m/z (%) 416 (M^+^, 9), 399 (13), 225 (21), 191 (100), 166 (12), 136 (87), 106 (36), 78 (49), 60 (11), 42 (40). Anal. Calcd. For C_17_H_16_N_6_O_5_S: C, 49.03; H, 3.87; N, 20.18; Found: C, 49.31; H, 3.58; N, 20.36.

#### 1-(2,6-difluorobenzyl)-4-(5-(5-nitrofuran-2-yl)-1,3,4-thiadiazol-2-yl)piperazine (6m)

Yield 50%; m.p. 176-179°C; IR (KBr): 1353, 1540 cm^-1^ (NO_2_); ^1^H-NMR(CDCl_3_) δ: 2.69 (t, 4H, piperazine, *J* = 4.6 Hz), 3.67 (t, 4H, piperazine, *J* = 4.6 Hz), 3.79 (s, 2H, CH_2_), 6.93 (t, 2H, phenyl, *J* = 7.4 Hz), 7.16 (d, 1H, furan, *J* = 3.8 Hz), 7.28-7.38 (m, 1H, phenyl), 7.43 (d, 1H, furan, *J* = 3.8 Hz); MS: m/z (%) 407 (M^+^, 5), 182 (85), 149 (11), 127 (100), 69 (11), 42 (11). Anal. Calcd. For C_17_H_15_F_2_N_5_O_3_S:C, 50.12; H, 3.71;N, 17.19; Found: C, 50.35; H, 3.45; N, 17.54.

#### 1-(2,4,5-trifluorobenzyl)-4-(5-(5-nitrofuran-2-yl)-1,3,4-thiadiazol-2-yl)piperazine (6n)

Yield 41%; m.p. 174-175°C; IR(KBr): 1355, 1517 cm^-1^ (NO_2_); ^1^H-NMR(CDCl_3_) δ: 2.65 (t, 4H, piperazine, *J* = 5.2 Hz), 3.60 (s, 2H, CH_2_), 3.68 (t, 4H, piperazine, *J* = 5.2 Hz), 6.84-6.86 (m, 1H, phenyl), 7.18 (d, 1H, furan, *J* = 3.8 Hz), 7.25-7.26 (m, 1H, phenyl), 7.74 (d, 1H, furan, *J* = 3.8 Hz); MS: m/z (%) 425 (M^+^, 6), 225 (13), 200 (94), 166 (11), 145 (100), 82 (12). Anal. Calcd. For C_17_H_14_F_3_N_5_O_3_S: C, 48.00; H, 3.32; N, 16.46; Found: C, 48.35; H, 3.44; N, 16.18.

#### 1-(2,5-dichlorobenzyl)-4-(5-(5-nitrofuran-2-yl)-1,3,4-thiadiazol-2-yl)piperazine (6o)

Yield 92%; m.p. 165-167°C; IR(KBr): 1353, 1549 cm^-1^ (NO_2_); ^1^H-NMR(CDCl_3_) δ: 2.71 (t, 4H, piperazine, *J* = 4.6 Hz), 3.67 (s, 2H, CH_2_), 3.68 (t, 4H, piperazine, *J* = 4.6 Hz), 7.17 (d, 1H, furan, *J* = 3.6 Hz), 7.22 (dd, 1H, phenyl, *J* = 8.5 Hz, *J* = 2.0 Hz), 7.32 (d, 1H, phenyl, *J* = 8.5 Hz, *J* = 2.0 Hz), 7.45 (d, 1H, furan, *J* = 3.6 Hz), 7.51 (d, 1H, phenyl, *J* = 2.0 Hz); MS: m/z (%) 443 (M^+^+4, 0.4), 441 (M^+^+2, 3), 439 (M^+^, 6), 313 (12), 236 (16), 214 (95), 192 (16), 159 (100), 123 (16), 99 (13), 82 (16), 57 (20), 40 (16). Anal. Calcd. For C_17_H_15_Cl_2_N_5_O_3_S: C, 46.37; H, 3.43; N, 15.91; Found: C, 46.65; H, 3.67; N, 15.69.

#### 1-(3,4-dichlorobenzyl)-4-(5-(5-nitrofuran-2-yl)-1,3,4-thiadiazol-2-yl)piperazine (6p)

Yield 62%; m.p. 161-162°C; IR(KBr): 1353, 1503 cm^-1^ (NO_2_); ^1^H-NMR(CDCl_3_) δ: 2.62 (t, 4H, piperazine, *J* = 5.0 Hz), 3.53 (s, 2H, CH_2_), 3.67 (t, 4H, piperazine, *J* = 5.0 Hz), 7.17 (d, 1H, furan, *J* = 3.8 Hz), 7.19 (dd, 1H, phenyl, *J* = 8.3 Hz, *J* = 2.0 Hz), 7.41-7.45(m, 2H, phenyl), 7.47 (d, 1H, furan, *J* = 3.8 Hz); MS: m/z (%) 443 (M^+^+4, 0.5), 441 (M^+^+2, 3), 439 (M^+^, 5), 273 (12), 238 (11), 214 (93), 159 (100), 123 (16), 100 (7), 82 (13), 56 (15). Anal. Calcd. For C_17_H_15_Cl_2_N_5_O_3_S: C, 46.37; H, 3.43; N, 15.91; Found: C, 46.68; H, 3.16; N, 16.09.

#### 1-(4-bromobenzyl)-4-(5-(5-nitrofuran-2-yl)-1,3,4-thiadiazol-2-yl)piperazine (6q)

Yield 28%; m.p. 192-193°C; IR(KBr): 1353, 1508 cm^-1^ (NO_2_); ^1^H-NMR(CDCl_3_) δ: 2.61 (t, 4H, piperazine, *J* = 5.0 Hz), 3.54 (s, 2H, CH_2_), 3.66 (t, 4H, piperazine, *J* = 5.0 Hz), 7.17 (d, 1H, furan, *J* = 3.9 Hz), 7.23 (d, 2H, phenyl, *J* = 8.3Hz), 7.44 (d, 1H, furan, *J* = 3.9 Hz), 8.25 (d, 2H, phenyl, *J* = 8.3 Hz); MS: m/z (%) 451 (M^+^+2, 1), 449 (M^+^, 2), 210 (20), 169 (100), 90 (44), 56 (22), 40 (21). Anal. Calcd. For C_17_H_16_BrN_5_O_3_S: C, 45.34; H, 3.58; N, 15.55; Found: C, 45.59; H, 3.92; N, 15.27.

## Biological activity

### Patients and bacterial strains

Different isolates of *H. pylori* were obtained from 160 dyspeptic patients consisted of 78 men and 82 women whose mean ages were 48 and 43 years, respectively. Based on the endoscopic diagnosis, the patients were classified into three groups: gastritis (124, 77.5%), ulcers (32, 20%) and cancer.

Antral biopsies demonstrating positive urease tests were transported to the microbiology lab in semisolid (0.1% agar) normal saline. The biopsies were cultured using selective medium containing brucella agar (Merck), 7% defibrinated sheep blood, vancomycin (5 mg/L), trimethoprim (5 mg/L), polymyxin B (50 mg/L), and amphotericin B (4 mg/L). Incubation of cultured isolates was performed at 37°C under microaerobic conditions (CO_2_ incubator; Heraeus, Germany). After 3–5 days, all cultures were examined for observation of pinpoint (1–2 mm) glistening colonies. Identification of *H. pylori* isolates was carried out according to the spiral microscopic appearance, Gram negative stain and some biochemical examinations such as urease, oxidase and catalase positive test and negative activities of nitrate and H_2_S.

The protocol of this research was approved by Pharmaceutical Sciences Research Center ethics committee (number 90-3-29: 1-1).

### Consent

Written informed consent was obtained from the patient for the publication of this report.

### Antimicrobial susceptibility test

Antimicrobial susceptibility test was performed using disk diffusion method (DDM). Recruited antibiotics included metronidazole, tetracycline. In the first step of the susceptibility evaluation, one hundred and ten strains were recruited. As a result of remarkable resistance of different studied bacterial isolates to recruited antibiotics and in attempt to increase the accuracy of the metronidazole resistant rates, in the second step of our study, an additional fifty strains of *H. Pylori* isolates were recruited for susceptibility testing with metronidazole (32, 16, 8, and 4 μg/mL) and with 2, 1, and 0.5 μg/mL of tetracycline. The susceptibility tests were repeated twice for the resistant strains. Bacterial suspensions were prepared in normal saline with the turbidity of Mac-Farland standard No.2 (equivalent to 6 × 108cell/mL). 100 μl of each bacterial suspension were inoculated in the surface of non- selective blood agar plates and the culture plates were allowed to dry at room temperature (10 min). Sterile blank disks were deposited on the surface of inoculated plates. 10 μL of each antibiotic dilution was poured into a blank disk. Moreover, control plates with growth positive bacterial culture were prepared using the introduction of 10 μl of the antibiotic solvent into the blank disks.

Plates were incubated at described condition and the inhibition zone diameters (IZD) were examined after 3–5 days. Susceptible and resistant isolates of *H.pylori* demonstrated IZDs ≥20 mm and ≤10 mm for metronidazole, respectively. The antibacterial activities of target compounds were evaluated against three metronidazole-resistant isolates of *H. pylori*. All experiments were performed in triplicate and the mean of IZDs produced by test compounds in four concentrations (100, 50, 25 and 12.5 μg/mL) was considered as antibacterial activity.

### Anti-Helicobacter pylori activity assay

As mentioned earlier, the growth inhibitory potential of test compounds was evaluated against three metronidazole resistant isolates of *H. pylori* by the filter paper disk diffusion method at 37°C, under microqerophilic condition on selective Brucella agar with 7% defibrinated horse blood. Four concentrations of titled compounds in dimethylsulfoxide (DMSO) were used for evaluation of anti *Helicobacter* activity assay. Blank standard disks (6 mm in diameter) were deposited on the surface of test plates and impregnated with 10 *μ*L of different concentrations of target compounds. Test plates were incubated at 37°C for 3–5 days and the inhibition zone around each disk (average diameter) was measured. The control disks were impregnated with 10 *μ*L of DMSO. All antibacterial activity experiments were performed in triplicate and the antibacterial activity was expressed as the mean of IZDs (mm) produced by the test compounds at each evaluated concentration.

## Result and discussion

### Chemistry

**Scheme 1:**
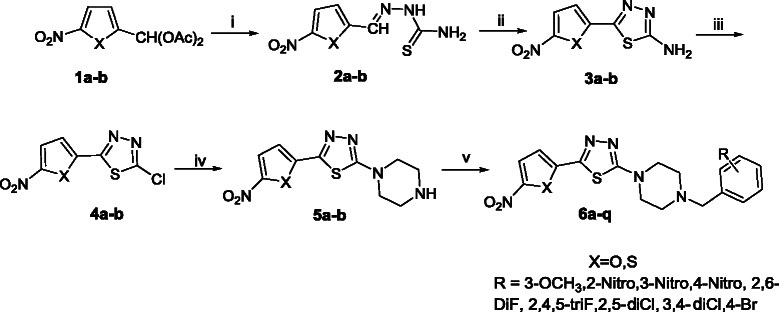
**Reagents and conditions: (i) thiosemicarbazide, EtOH, HCl, reflux, 1.5 h; (ii) NH**_**4**_**Fe(SO**_**4**_**)**_**2**_**, 12H**_**2**_**O, H**_**2**_**O reflux, 25 h; (iii) NaNO**_**2**_**, HCl, Cu, °C→ rt, 3 h; (iv) Piperazine hydrate, EtOH, NaHCO**_**3 **_**1 h; (v) DMF, Substituted benzyl chloride, 4 h.**

The synthetic pathway for the target compounds **6a-q** is depicted in the Scheme 1. A mixture of 5-nitroaryl-2-carboxaldehyde diacetate **1a-b** with thiosemicarbazide was refluxed in ethanol to afford thiosemicarbazone **2a-b**. Amino-1,3,4-thiadiazoles **3a-b,** were synthesized through the oxidative cyclization of **2a-b** in presence of ammonium ferric sulfate. In the next step, diazotization of **3a-b** in hydrochloric acid and in the presence of copper powder yielded chloro-1,3,4-thiadiazole **4a-b**. 1-(5-(5-nitroaryl-2-yl)-1,3,4-thiadiazol-2-yl)piperazine **5a-b** were prepared through the reaction of chloro-1,3,4-thiadiazole derivatives **4a-b** with piperazine hydrate in stirred ethanol.

**Figure 2:**
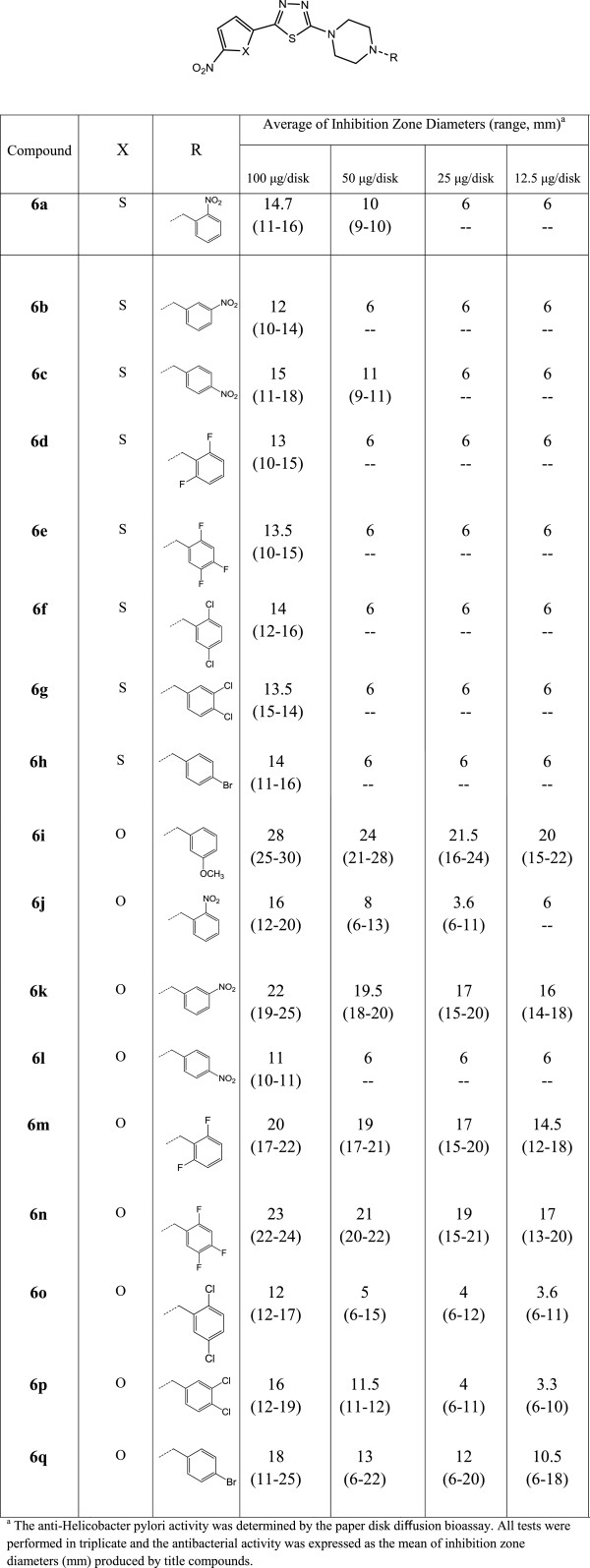
**Average of inhibition zone diameters of compounds 6a-q at four different concentrations against three metronidazole resistant *H. pylori *isolates.**

The prepared key intermediates **5a-b** were further reacted with different substituted benzyl chlorides in refluxing DMF to give the corresponding 1-substituted-benzyl-4-(5-(5-nitroaryl-2-yl)-1,3,4-thiadiazol-2-yl)piperazine **6a-q**. The structures of compounds **6a-q** were determined using spectroscopic methods including mass spectrometry,^1^H NMR, IR, and elemental analysis. The chemical structure of target compounds are shown in Figure 2.

### Anti-*Helicobacter pylori* activity and structure-activity relationship study

The *in vitro* anti-Helicobacter activity of synthesized derivatives was determined by paper disk diffusion bioassay against three metronidazole resistant *H. pylori* isolates. The average of inhibition zone diameters (IZD) of compounds against three isolates at four different concentrations (100, 50, 25 and 12.5 μg/ disk) is summarized in Figure 2. The anti-*H. pylori* activity of target derivatives could be simply categorized as follows: strong response, zones range diameter *>*20 mm; moderate response, zone diameter 16–20 mm; weak response, zone diameter 11–15 mm; and little or no response, zone diameter *<*10 mm [15].

Investigation of the IZD of studied compounds revealed that the target derivatives demonstrated a wide spectrum of anti-*H. pylori* activity varied from little (IZD <10 mm) to strong (IZD >20 mm) response at concentration of 100 μg/disk against metronidazole resistant strains. In view of the obtained data, the following structure-activity relationship might be developed:

### Assessment of nitroheterocyclic moiety

Based on the substituted nitroaromatic group, the studied 1,3,4-thiadiazole derivatives could be classified into two groups: Nitrothiophen **6a-h** and nitrofuran **6i-q** derivatives. The results of anti-*H. pylori* activity indicated that the inhibitory responses of test compounds is mainly attributed to the substituted nitroaryl moiety at the C-5 position of 1,3,4-thiadiazole ring. While all of nitrothiophene derivatives **6a-h** demonstrated weak (IZD = 11–15 mm) to little (IZD <10 mm) inhibitory response at concentration of 100 μg/disk against three metronidazole resistant isolates, most of nitrofurane derivatives **6i-q** showed strong (IZD > 20 mm) to moderate (IZD = 16–20) growth inhibitory potential at the same concentration.

It could be concluded that nitrofuran **6i-q** derivatives of 1,3,4-thiadiazole scaffold, are more potent than the nitrothiophen **6a-h** counterpart.

#### Investigation of substituted group into the benzyl piperazine pendant

In order to find the structural requirement of substituted moiety at C-2 position of 1,3,4-thiadiazole scaffold, different benzyl piperazine derivatives were substituted at the described position. Among the nitrofuran derivatives **6i-q**, 3-methoxybenzyl piperazine derivative **6i,** demonstrated strong anti-*H. pylori* potential at studied concentrations 100–25 μg/disk (IZD > 20 mm) against studied isolates. Investigation of the growth inhibitory potential of the nitrofuran series **6i-q** revealed that substitution of nitro group at the *meta* position of the benzyl piperazine side chain, resulted in compound with strong (IZD = 20 mm) to moderate (IZD = 16–20 mm) growth inhibitory potential at 100 and 50–12.5 μg/disk, respectively. However, introduction of nitro substitute at *ortho* or *para* position of the benzyl piperazine pendant, resulted in compound with diminished inhibitory potential against resistant strains of *H. pylori* isolates (compounds **6j** (IZD = 16 mm, moderate response) and **6l** (IZD = 11 mm, weak response) respectively). Moreover, substitution of fluorine groups at different positions of benzyl pieperzine side chain influenced the growth inhibitory potential of compounds which is mainly dependent on the position and number of substituted fluorine groups; compound **6n** containing 2,4,5-triflouro benzyl piperazine pendant at C-2 position of 5-nitrofuran-1,3,4-thiadiazole scaffold, produced strong inhibitory response at 100 and 50 μg/disk (IZD =23 and 21 mm, respectively); while the anti-*H. pylori* potential of 2,5-difluoro benzyl piperazine counterpart was diminished to weak (IZD = 12 mm) to no response (IZD = 5 mm) at 100 and 50 μg/disk, respectively.

## Conclusion

A novel series of 5-(5-nitroaryl)-1,3,4-thiadiazole derivatives containing various benzyl piperazine moiety at C-2 position of 1,3,4-thiadiazole ring were synthesized and evaluated against three metronidazole-resistant isolates of *H.pylori* using paper disk diffusion bioassay test. Structure-activity relationship study of these derivatives indicated that 1,3,4-thiadiazole derivatives bearing 5-nitrofuran moiety at C-5 position of central thiadiazole ring, demonstrated more promising anti-*H. pylori* than the 5-nitrothiophen counterpart. The most potent nitrofuran derivative had 3-methoxybenzyl piperazine pendant at the C-2 position of 1,3,4-thiadiazole ring. The results indicated that the anti-*H. pylori* potential of the nitrofurane derivatives of 1,3,4-thiadiazole scaffold is mainly attributed to the type and position of the substituted group at the benzyl piperazine pendant. Future studies may be aimed at designing more potent derivatives of these series in order to investigate the structure-activity relationship of cyclic amine derivatives of 5-(nitroaryl)-1,3,4-thiadiazole derivatives as Anti-*H. pylori* agents.

## Competing interests

The authors declare that they have no competing interests.

## Authors’ contributions

NM: Synthesis of some target compounds. PS: Evaluation of the antibacterial activities (10%). AG: Evaluation of the antibacterial activities. HA: Evaluation of the antibacterial activities (10%). FA: Synthesis of the intermediates and some target compounds. NE: Collaboration in identifying of the structures of target compounds. FS: Evaluation of the antibacterial activities. AF: Collaboration in design and identifying of the structures of target compounds, manuscript preparation. AS: Design of target compounds and management of the synthetic and pharmacological parts. All authors read and approved the final manuscript.
